# How useful are hemoglobin concentration and its variations to predict significant hemorrhage in the early phase of trauma? A multicentric cohort study

**DOI:** 10.1186/s13613-018-0420-8

**Published:** 2018-07-06

**Authors:** S. Figueiredo, C. Taconet, A. Harrois, S. Hamada, T. Gauss, M. Raux, J. Duranteau, the Traumabase Group, Arie Attias, Sylvain Ausset, Mathieu Boutonnet, Gilles Dhonneur, Olivier Langeron, Catherine Paugam-Burtz, Romain Pirracchio, Bruno Riou, Guillaume de St Maurice, Bernard Vigué

**Affiliations:** 1 Department of Anaesthesia and Critical Care, Assistance Publique – Hôpitaux de Paris, Hôpital Bicêtre, University Paris-Sud, 78 rue du Général Leclerc, 94275 Le Kremlin Bicêtre, France; 2 Hôpitaux Universitaires Paris Sud, 94275 Le Kremlin Bicêtre, France; 3 Department of Anaesthesia and Critical Care, Beaujon Hospital, Hôpitaux Universitaires Paris-Nord Val-de-Seine, Assistance Publique – Hôpitaux de Paris, Clichy, France; 4 SSPI - Accueil des Polytraumatisés, Hôpital Universitaire Pitié Salpêtrière - Charles Foix, Assistance Publique – Hôpitaux de Paris, Paris, France; 5 INSERM UMR_S 1158 Neurophysiologie Respiratoire Expérimentale et Clinique, Université Pierre et Marie Curie, Paris, France

**Keywords:** Hemorrhage, Hemoglobin, Trauma, Point-of-care systems, Resuscitation

## Abstract

**Background:**

The diagnostic value of hemoglobin (Hb) for detecting a significant hemorrhage (SH) in the early phase of trauma remains controversial. The present study aimed to assess the abilities of Hb measurements taken at different times throughout trauma management to identify patients with SH.

**Methods:**

All consecutive adult trauma patients directly admitted to six French level-1 trauma centers with at least one prehospital Hb measurement were analyzed. The abilities of the following variables to identify SH (≥ 4 units of red blood cells in the first 6 h and/or death related to uncontrolled bleeding within 24 h) were determined and compared to that of shock index (SI): Hb as measured with a point-of-care (POC) device by the prehospital team on scene (POC-Hb_prehosp_) and upon patient’s admission to the hospital (POC-Hb_hosp_), the difference between POC-Hb_hosp_ and POC-Hb_prehosp_ (DeltaPOC-Hb) and Hb as measured by the hospital laboratory on admission (Hb-Lab_hosp_).

**Results:**

A total of 6402 patients were included, 755 with SH and 5647 controls (CL). POC-Hb_prehosp_ significantly predicted SH with an area under ROC curve (AUC) of 0.72 and best cutoff values of 12 g/dl for women and 13 g/dl for men. POC-Hb_prehosp_ < 12 g/dl had 90% specificity to predict of SH. POC-Hb_hosp_ and Hb-Lab_hosp_ (AUCs of 0.92 and 0.89, respectively) predicted SH better than SI (AUC = 0.77, *p* < 0.001); best cutoff values of POC-Hb_hosp_ were 10 g/dl for women and 12 g/dl for men. DeltaPOC-Hb also predicted SH with an AUC of 0.77, a best cutoff value of − 2 g/dl irrespective of the gender. For a same prehospital fluid volume infused, DeltaPOC-Hb was significantly larger in patients with significant hemorrhage than in controls.

**Conclusions:**

Challenging the classical idea that early Hb measurement is not meaningful in predicting SH, POC-Hb_prehosp_ was able, albeit modestly, to predict significant hemorrhage. POC-Hb_hosp_ had a greater ability to predict SH when compared to shock index. For a given prehospital fluid volume infused, the magnitude of the Hb drop was significantly higher in patients with significant hemorrhage than in controls.

**Electronic supplementary material:**

The online version of this article (10.1186/s13613-018-0420-8) contains supplementary material, which is available to authorized users.

## Background

Hemorrhage is the leading cause of death following trauma, either quickly by exsanguination or later by organ failure following hypoperfusion [1, 2]. Early detection of severe hemorrhage during the prehospital phase and immediately at the admission of trauma patients is crucial to initiate appropriate resuscitation and to trigger subsequent lifesaving interventions such as massive transfusion and/or hemostatic therapy (surgery or angioembolization) [3]. Low hemoglobin (Hb) or hematocrit (Hct) values are widely and interchangeably used as indicators of severe bleeding [3]. However, the diagnostic value of Hb or Hct for detecting hemorrhage in trauma patients at the initial phase remains controversial [4–14]. So far, no study has evaluated the diagnostic value of early Hb measurements obtained by point-of-care (POC) devices in the prehospital setting and immediately after hospital admission. These POC devices provide a rapid and minimally invasive Hb measurement [15] and are routinely used by prehospital and hospital teams within the trauma network of the Paris Ile-de-France area (France).

The aim of the present study was to answer to the following questions: (1) What are the abilities of the following Hb-based variables to predict significant hemorrhage (SH): the first Hb measurements taken using a POC device on scene (POC-Hb_prehosp_) and at hospital admission (POC-Hb_hosp_), the difference between hospital and prehospital POC-Hb values (DeltaPOC-Hb) and the first Hb level provided by the hospital laboratory on admission (Hb-Lab_hosp_)? (2) Are those parameters more reliable than the widely used shock index (SI = heart rate/systolic arterial pressure) [16, 17] in predicting SH?

## Methods

### Study design and setting

This retrospective cohort study was conducted between November 2011 and July 2016 in six French academic (level-1) trauma centers in Paris area, France: Beaujon, Bicêtre, Henri Mondor, Georges Pompidou, Percy and Pitié-Salpêtrière Hospitals. The institutional review board (“Comité de Protection des Personnes Paris Ile-de-France VI,” Paris, France) gave its approval and waived the need for written informed consent. The study was performed by using the TraumaBase^®^, a prospective trauma registry that includes all trauma patients admitted to the six centers involved in this study. In accordance with the French Law, the registry was approved by the Advisory Committee for Information Processing in Health Research (Comité Consultatif sur le Traitement de l’Information en matière de Recherche dans le domaine de la Santé, N° 11.305 bis) and the French National Commission on Computing and Liberty (Commission Nationale Informatique et Liberté, n°911461). Since 2010, the clinical files of all trauma patients were standardized in every participating hospital to allow a reproducible and homogenous data collection including prehospital and hospital items. Although data were prospectively acquired in the TraumaBase^®^ for research and epidemiological purposes, the present study should be considered as a retrospective study.

### Trauma organization in the Paris Ile-de-France Region

In France, emergency calls are centralized to the phone number “15.” Depending on the information provided by those calls, a 24/7 available dispatching physician decides whether a paramedic-staffed ambulance or a physician-staffed mobile intensive care unit (Service Mobile d’Urgence et de Réanimation [SMUR]) is to be deployed. If a major trauma is suspected, a SMUR vehicle is deployed. Major trauma is usually suspected by using the French national Vittel triage criteria [18, 19], which provide a prehospital five-step evaluation of the trauma severity including physiologic, anatomic and resuscitation parameters as well as global assessment of speed and mechanism. The presence of one or more of the 26 criteria usually leads to a level-1 trauma center admission. The prehospital medical team performs clinical evaluation on scene, initiates prehospital resuscitation and carries the trauma patient to the receiving hospital.

### Selection of participants

All consecutive adult (18 years and older) trauma patients directly admitted from the trauma scene to one of the six aforementioned level-1 trauma centers for suspicion of major trauma were included in the study if they had at least one prehospital Hb measurement with the POC HemoCue^®^ device (Hb201, Ångelholm, Sweden). This Hb measurement is routinely performed by the SMUR team at arrival on scene and by the hospital team at hospital admission.

### Measurements

For each patient admitted to a study center, the following data were recorded: age, sex, mechanism of injury, prehospital lowest systolic arterial blood pressure (BP), highest heart rate (HR), Glasgow coma scale score, amount of prehospital fluid volume administered (*FV*_*prehosp*_), time from the emergency call to the SMUR team arrival on scene, prehospital care time, vital signs on arrival, injury severity score (ISS), biological variables (including Hb and serum lactate measurements), transfusion therapy within the first 24 h of injury, hemostatic therapy (angioembolization and surgery) and outcome variables (mortality, intensive care unit (ICU) length of stay and duration of mechanical ventilation). Serum lactate (*lactate*_*hosp*_) was measured at hospital admission either in the laboratory or using a point-of-care blood gases analyzer (ABL 800, Radiometer). Prehospital and hospital shock index (*SI*_*prehosp*_ and *SI*_*hosp*_, respectively) values were calculated using the following formulas, respectively: maximal HR_prehosp_/minimal systolic BP_prehosp_ and HR at hospital admission/systolic BP at hospital admission.

### Outcome and definitions

The primary outcome of interest was the occurrence of *significant hemorrhage* (SH), defined as the need for transfusion of at least 4 units of packed RBCs in the first 6 h and/or death related to uncontrolled bleeding in the first 24 h following injury. Patients without SH were considered as *controls* (CL). The decision to transfuse was taken by the physician in charge of the patient at the trauma center, based on his evaluation of the patient’s clinical situation (clinical signs of shock, positive FAST (focused assessment with sonography for trauma) echography or obvious external hemorrhage).

The first Hb measurement taken using a POC device on scene is referred to as *POC*-*Hb*_*prehosp*_ and the first one performed at hospital admission as *POC*-*Hb*_*hosp*_. The difference between hospital and prehospital POC-Hb values was calculated (*POC*-*Hb*_*hosp*_ − *POC*-*Hb*_*prehosp*_) and named *DeltaPOC*-*Hb*. Hb measured in the laboratory upon admission to the hospital is referred to as *Hb*-*Lab*_*hosp*_.

### Statistical analysis

Continuous data are expressed as mean ± standard deviation or median [quartile 1; 3] according to their distribution (graphical plotting assessment). Categorical data are expressed as counts and percentages. Comparisons were made using the Student t test or the nonparametric Mann–Whitney *U* test, depending on the normality of the data. The comparison of proportions was made using Chi-squared test or Fisher’s exact test when appropriate.

The abilities of POC-Hb_prehosp_, POC-Hb_hosp_, DeltaPOC-Hb and Hb-Lab_hosp_ to predict SH were first tested using univariate logistic regression. In addition, the abilities of FV_prehosp_, lactate_hosp_, SI_prehosp_ and SI_hosp_ to predict SH were also tested using univariate logistic regression. Sensitivity, specificity, positive and negative predictive values and calculation of areas under the curve (AUC and 95% confidence interval) were calculated for each variable. When the AUC was greater than 0.5, the Youden index was calculated (sensitivity + specificity − 1), and the value for the maximum of the Youden index was considered as the best cutoff point.

AUCs of the prehospital and hospital variables were, respectively, compared to the corresponding AUCs of SI_prehosp_ and SI_hosp_ using the Hanley–McNeil test.

Since the binary approach of the ROC curve analysis is not always an adequate representation of the clinical reality, a gray zone approach was performed as described elsewhere [21, 22]. First, ROC curves were obtained by averaging 2000 populations bootstrapped (sampling with replacement) from the original study population to determine 95% CI of the best thresholds of the bootstrap population for each variable. A second step was performed to define a threshold related to a sensitivity of 90% and another threshold related to a specificity of 90% using the 2000 populations. In accordance with the original publication of the gray zone approach [21] the widest obtained gray zone interval obtained by one of these methods was selected as the final gray zone.

Since women and men have different baseline Hb levels [20], a gender-based analysis was performed for each Hb-related variable (POC-Hb_prehosp_, POC-Hb_hosp_, DeltaPOC-Hb and Hb-Lab_hosp_) to determine the AUCs and/or best cutoff values of these variables more accurately in female and male patients.

A multivariate logistic regression gathering all predictive variables available at each prehospital and hospital period was performed to assess the usefulness of each variable in severe hemorrhage prediction.

In addition, interactions between DeltaPOC-Hb and the amount of prehospital fluid volume administered for patients with significant hemorrhage and controls were analyzed using a multiple linear model (checked for residuals) to specifically test the hypothesis that the presence of significant hemorrhage can affect the effect of prehospital fluid volume on the drop of Hb level between prehospital and hospital phases. The interaction term (FV_prehosp_ * SH) was also assessed.

All *p* values were two-tailed, and a value of *p* < 0.05 was considered significant. Analyses were performed using JMP^®^, version 9 (SAS Institute Inc., Cary, NC), MedCalc 7.3.0.1 software (Mariakerke, Belgium) and R 3.3.3 (http://www.R-project.org/) packages.

## Results

### Characteristics of study subjects

**Fig. 1:**
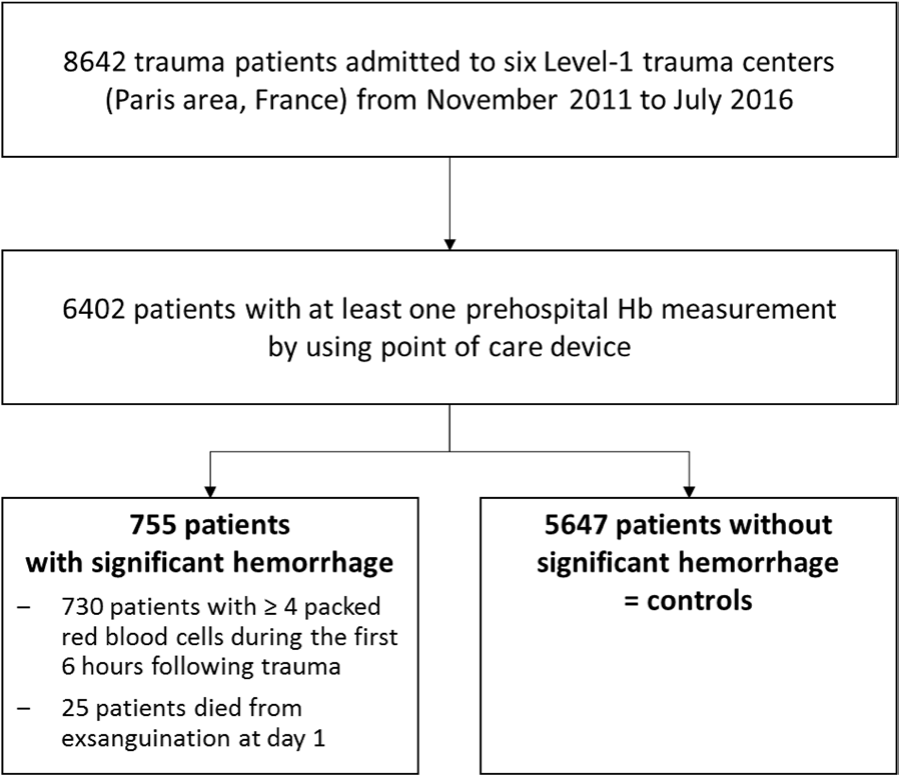
Flow diagram of the study

During the study period, 8642 consecutive trauma patients were directly admitted from trauma scene to the six trauma centers, of which 6402 had at least one POC-Hb_prehosp_ (74% of the cohort). A total of 755 (12%) patients had SH (including 25 patients who died from uncontrolled bleeding during the first 24 h), and 5647 patients were controls (CL) without SH (Fig. 1). The main characteristics of the patients are presented in Table 1. Patients with SH had higher ISS, more transfusion requirement at any time and higher ICU mortality (37% vs. 7%, *p* < 0.001) when compared to controls.

**Table 1:** General characteristics for patients with significant hemorrhage and controls

	SH *n* = 755	CL *n* = 5647	*p* value
Age (years)	38 [25–55]	33[24–47]	< 0.001
Male gender (*n*, %)	538 (71)	4490 (80)	< 0.001
BMI (kg cm^−2^)	24 [22–27]	24 [22–27]	NS
ISS	33 [20–43]	11 [5–20]	< 0.001
Blunt (*n*, %)	656 (87)	5020 (89)	NS
Time from the emergency call to the EMS arrival on trauma scene (min)	10 [6–14]	10 [6–15]	NS
Minimal systolic BP_prehosp_ (mm Hg)	80 [60–99]	118 [102–130]	< 0.001
Maximal HR_prehosp_ (bpm)	106 [80–126]	90 [80–106]	0.056
Shock index_prehosp_	1.2 [0.8–1.5]	0.8 [0.6–0.95]	< 0.001
FV_prehosp_ total (ml)	1500 [1000–2000]	500 [500–1000]	< 0.001
FV_prehosp_ crystalloids (ml)	1000 [500–1500]	500 [500–1000]	< 0.001
FV_prehosp_ colloids (ml)	0 [0–500]	0 [0–0]	< 0.001
Prehospital care time (min)	80 [60–104]	71 [53–97]	< 0.001
HR at admission (bpm)	102 [80–120]	86 [74–100]	< 0.001
Systolic BP at admission (mm Hg)	96 [74–117]	129 [114–143]	< 0.001
Diastolic BP at admission (mm Hg)	76 [66–87]	56 [42–72]	< 0.001
Shock index_hosp_	1 [0.75–1.4]	0.7 [0.6–0.8]	< 0.001
Lactate_hosp_ (mmol liter^−1^)	4.3 [3–8]	2.8 [1–3]	< 0.001
Platelets (10^3^ mm ^−3^)	171 [117–221]	225 [189–264]	< 0.001
Prothrombin time (quick %)	48 [32–63]	84 [73–93]	< 0.001
Fibrinogen (g liter^−1^)	1.3 [0.9–1.8]	2.4 [2–2.8]	< 0.001
*Transfusion requirement*			
RBCs (U) at H1	3 [1–4]	0 [0–0]	< 0.001
FFP (U) at H1	0 [0–3]	0 [0–0]	< 0.001
PLT (PC) at H1	0 [0–0]	0 [0–0]	< 0.001
RBCs (U) at H6	6 [4–8]	0 [0–0]	< 0.001
FFP (U) at H6	4 [3–7]	0 [0–0]	< 0.001
PLT (PC) at H6	0 [0–1]	0 [0–0]	< 0.001
RBCs (U) at H24	7 [4–10]	0 [0–0]	< 0.001
FFP (U) at H24	6 [3–9]	0 [0–0]	< 0.001
PLT (PC) at H24	1 [0–1]	0 [0–0]	< 0.001
IGS II score	52 [37–72]	16 [9–29]	< 0.001
ICU mortality (*n*, %)	275 (37)	364 (7)	< 0.001

*BMI* body mass index, *BP* arterial blood pressure, *CL* control, *EMS* emergency medical service, *FFP* fresh frozen plasma, *FV*_*prehosp*_ prehospital fluid volume, *H1* one hour after hospital admission, *H6* six hour after trauma, *H24* twenty-four hour after trauma, *HR* heart rate, *ISS* injury severity score, *lactate*_*hosp*_ serum lactate at the admission to hospital, *NS* nonsignificant, *PC* platelet concentrates, *RBCs* red blood cells, *SH* significant hemorrhage, *shock index*_*prehosp*_ maximal HR_prehosp_/minimal systolic arterial BP_prehosp_, *shock index*_*hosp*_ HR at admission/systolic arterial BP at admission, *U* units

### Main results

Data on Hb measurements are presented in Table 2. All Hb-derived measures were significantly different between SH and CL groups (*p* < 0.001) (Table 2).

**Table 2:** Different hemoglobin measurements and variations

	SH *n* = 755	CL *n* = 5647	*p* value
POC-Hb_prehosp_ (g dl^−1^)	12.5 [11–14]	14.0 [13–15]	< 0.001
POC-Hb_hosp_ (g dl^−1^)	9.6 [8–11]	13.5 [12–15]	< 0.001
DeltaPOC-Hb (g dl^−1^)	− 3 [− 5;− 1]	− 1 [− 2;0]	< 0.001
Hb-Lab_hosp_ (g dl^−1^)	9.3 [7.6–11]	13.5 [12–14.5]	< 0.001

*SH* significant hemorrhage, *CL* controls, *POC-Hb*_*prehosp*_ *and POC-Hb*_*hosp*_ prehospital and hospital hemoglobin levels provided by point-of-care device, *DeltaPOC-Hb* POC-Hb_hosp_ − POC-Hb_prehosp_, *Hb-Lab*_*hosp*_ hemoglobin level provided by the laboratory at hospital admission

**Fig. 2:**
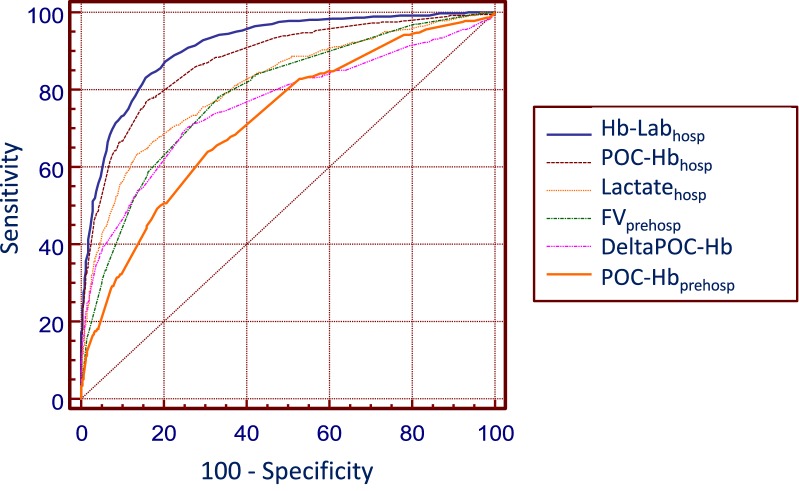
Receiver operating characteristic (ROC) curves describing the abilities of different parameters to predict significant hemorrhage. *Hb-Lab*_*hosp*_ hemoglobin level provided by the laboratory at hospital admission, *POC-Hb*_*prehosp*_ *and POC-Hb*_*hosp*_ prehospital and hospital hemoglobin levels provided by the point-of-care (POC) HemoCue^®^ device, *DeltaPOC-Hb* POC-Hb_hosp_ − POC-Hb_prehosp_, *FV*_*prehosp*_ prehospital fluid volume infused, *lactate*_*hosp*_ serum lactate measured at hospital admission

The abilities of the different variables to predict significant hemorrhage (SH) are presented in Table 3 and Fig. 2. During the prehospital phase, POC-Hb_prehosp_, prehospital fluid volume (FV_prehosp_) and prehospital shock index (SI_prehosp_) significantly predicted SH with respective AUCs of 0.72, 0.79 and 0.71. The prediction of SH by using the POC-Hb_prehosp_ cutoff value of 13 g/dl was appropriate in 69% of the patients (7.5% true positive and 61.4% true negative). AUC of POC-Hb_prehosp_ was not different from that of SI_prehosp_ (*p* = 0.44), whereas AUC of FV_prehosp_ was statistically higher than AUC of SI_prehosp_ (*p* < 0.001). The multivariate logistic regression performed on prehospital variables showed that SI_prehosp_, POC-Hb_prehosp_ and FV_prehosp_ were independently associated with SH (Additional file 1: Table 1).

**Table 3:** Predictive performances of different variables for significant hemorrhage

Variable	AUC [IC95%]	Cutoff	Se (%)	Spe (%)	PLR (%)	NLR (%)	PPV (%)	NPV (%)	Y	Gray zone (range)
Shock index_prehosp_	0.71 [0.70–0.72]	1	62	83	3.6	0.5	32	94	0.45	0.1–1.2
POC-Hb_prehosp_ (g dl^−1^)	0.72 [0.71–.73]	13	64	70	2.1	0.5	22	94	0.34	12– 15
FV_prehosp_ (ml)	0.79 [0.78–0.80]	900	78	68	2.4	0.3	25	96	0.46	500–1500
Shock index_hosp_	0.77 [0.76–0.78]	0.9	62	86	4.4	0.4	36	95	0.48	0.5–1
POC-Hb_hosp_ (g dl^−1^)	0.88 [0.88–0.89]	11.4	78	84	4.9	0.3	40	97	0.62	11–13
DeltaPOC-Hb (g dl^−1^)	0.77 [0.76–0.78]	− 2	70	77	3.0	0.4	29	95	0.46	− 3–0
Lactate_hosp_ (mmol liter^−1^)	0.81 [0.80–0.83]	3.5	63	87	4.7	0.4	41	94	0.49	2–4
Hb-Lab_hosp_ (g dl^−1^)	0.92 [0.91–0.92]	11.8	88	81	4.5	0.2	37	98	0.68	11–12

Shock index_prehosp_ = maximal HR_prehosp_/minimal systolic arterial BP_prehosp_; POC-Hb_prehosp_ prehospital hemoglobin level provided by the point-of-care HemoCue^®^ device; FV_prehosp_ prehospital fluid volume infused; POC-Hb_hosp_ hemoglobin level provided by the point-of-care HemoCue^®^ device at hospital admission; DeltaPOC-Hb = POC-Hb_hosp_ - POC-Hb_prehosp_; Hb-Lab_hosp_ hemoglobin level provided by the laboratory at hospital admission; shock index_hosp_ = HR at admission/systolic arterial BP at admission

*AUC* area under the ROC curve, *Se* sensitivity, *Spe* specificity, *PLR* positive likelihood ratio, *NLR* negative likelihood ratio, *POC* point-of-care, *PPV* positive predictive value, *NPV* negative predictive value, *Y* Youden

POC-Hb_hosp_ and Hb-Lab_hosp_, performed after hospital admission, had the best predictive performances for SH with AUCs of 0.88 and 0.92, with best cutoff values of 11.8 and 11.4 g/dl, respectively. Regarding the other variables measured at hospital admission: DeltaPOC-Hb, lactate_hosp_ and shock index at hospital admission (SI_hosp_) showed similar abilities to predict SH, with AUCs of 0.77, 0.81 and 0.77, with best cutoff values of − 2 g/dl, 3.5 mmol/l and 0.9, respectively. The multivariate logistic regression performed on hospital variables showed that they were all independently associated with SH, except DeltaPOC-Hb (*p* = 0.06) (Additional file 1 Table 2).

The borders of the gray zone for each predictive variable are presented in Table 3. For all variables, the widest gray zone corresponded to the zone between the thresholds related to 90% specificity (lower limit) and 90% sensitivity (upper limit).

Gender-based analysis showed that AUCs of each Hb-related variable (POC-Hb_prehosp_, POC-Hb_hosp_, DeltaPOC-Hb and Hb-Lab_hosp_) were similar between men and women (data not shown). Best cutoff values of POC-Hb_prehosp_, POC-Hb_hosp_ and Hb-Lab_hosp_ were lower in women than in men, respectively: 12 vs 13 g/dl for POC-Hb_prehosp_, 10 vs 12 g/dl for POC-Hb_hosp_ and 10 vs 11.8 g/dl for Hb-Lab_hosp_. Best cutoff value of DeltaPOC-Hb for the prediction of SH was the same (− 2 g/dl) for men and women.

**Fig. 3:**
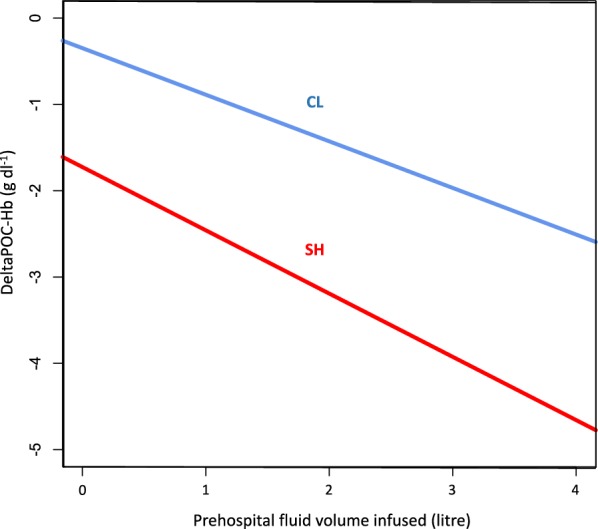
Relationship between the Hb drop from prehospital phase to hospital admission measured by POC device (DeltaPOC-Hb = POC-Hb_hosp_ − POC-Hb_prehosp_) and the prehospital fluid volume (FV_prehosp_) infused according to the level of bleeding (LB): significant hemorrhage (SH) or controls (CL). Multivariate linear regression without interaction term: $$\begin{array}{*{20}l} {{\text{DeltaPOC-Hb}}= - 3 + \left( { - 0.6} \right) \times {\text{FV}}_{\text{prehosp}} + \left( { - 1.6} \right) \times {\text{LB}}} \hfill \\ {{\text{FV}}_{\text{prehosp}}:\, p < 10^{ - 15} . \, {\text{ LB }}{:} \, p < 10^{ - 15} .{\text{ LB }} = \, 0\;{\text{ for}}\;{\text{CL}},{\text{ LB}} = 1\;{\text{ for}}\;{\text{SH}}.} \hfill \\ \end{array}$$ Multivariate linear regression with interaction term: $$\begin{array}{*{20}l} {{\text{DeltaPOC-Hb}}= - 0.35 + \left( { - 0.5} \right) \times {\text{FV}}_{\text{prehosp}} + \left( { - 1.4} \right) \times {\text{LB}} + \left( { - 0.2} \right) \times {\text{FV}}_{\text{prehosp}} {\text{LB}}} \hfill \\ {{\text{FV}}_{\text{prehosp}}: \, p < 10^{ - 15} .{\text{ LB}}: \, p < 10^{ - 15} .{\text{ Interaction}}\;{\text{term FV}}_{\text{prehosp}} *{\text{LB:}} \, p = 0.03.} \hfill \\ {{\text{LB}} = 0\;{\text{ for}}\;{\text{ CL}},\;{\text{LB}} = 1\;{\text{ for}}\;{\text{ SH}}.} \hfill \\ \end{array}$$ For the same prehospital fluid volume infused, the Hb drop from prehospital phase to hospital admission measured by POC device (DeltaPOC-Hb) was larger in patients with SH than in CL

The multiple linear regression on DeltaPOC-Hb found that FV_prehosp_ and SH (present or absent) were independently associated with DeltaPOC-Hb (*p* < 10^−15^). Moreover, the interaction between SH and FV_prehosp_ was also significant (*p* = 0.03). As shown in Fig. 3, the slopes of the regressions lines between DeltaPOC-Hb and FV_prehosp_ were significantly different between patients with significant hemorrhage and controls. Thus, for the same prehospital fluid volume infused, the drop in Hb as measured using the POC device (represented by DeltaPOC-Hb) was larger in patients with significant hemorrhage than in controls.

## Discussion

In 6402 consecutive trauma patients, the present study showed the main following findings:

(1) POC-Hb_prehosp_, the first Hb measurement taken on scene using a POC device, significantly and independently predicted significant hemorrhage (SH), with an AUC of 0.72 and best cutoff value of 12 g/dl for women and 13 g/dl for men. Thus, POC-Hb_prehosp_ could represent an additional tool for early identification of SH and subsequent implementation of adequate resuscitative and hemostatic therapies; (2) POC-Hb_hosp_, a quick Hb measurement performed on the trauma bay at hospital admission by using the same POC device, had a better ability to identify patients with SH (AUC of 0.88 and best cutoff values of 10 g/dl for women and 12 g/dl for men) than the widely used shock index. The diagnostic performance of this immediately available variable was equivalent to that of Hb-Lab_hosp_, the Hb value measured in the hospital laboratory (and so results are subject to delay); (3) DeltaPOC-Hb, the difference between hospital and prehospital POC-Hb values, significantly predicted SH (AUC of 0.77) with the same best cutoff value of − 2 g/dl in both men and women; (4) for a given prehospital fluid volume infused, the magnitude of the Hb drop was significantly higher in patients with SH than in controls.

As far as we know, this is the first report evaluating the diagnostic value of prehospital Hb measurement in predicting SH. All previously published studies evaluated Hb or Hct (these two variables being widely and interchangeably used as indicators of severe bleeding [3]) at hospital admission [4–14]. Interestingly, POC-Hb_prehosp_ obtained on scene by the prehospital team (median time from alert to arrival on scene was 10 min) was significantly lower in patients who developed SH (median 12.5 g/dl) compared to patients who did not (CL; median 14 g/dl). The gray zone approach determined that a POC-Hb_prehosp_ < 12 g/dl had 90% specificity to predict SH.

The fair ability, albeit modest, of POC-Hb_prehosp_ to predict SH challenges the conception that Hb and Hct in the early phase of hemorrhage are of little value because “the patient bleeds whole blood” and compensatory mechanisms for hypovolemia (transcapillary refill and fluid resuscitation) have not yet occurred. Data obtained from both animals and humans also showed that a drop in Hb/Hct might occur rapidly (within ten minutes) after the initiation of hemorrhage [18, 19]. As a consequence, the prehospital Hb measurement taken by using a POC device might be useful in alerting hospital teams to the possible need for transfusion or hemostatic therapy. This is a major finding of this study, and further prospective studies are required to evaluate the clinical impact of the use of this variable, alone or in combination with other ones.

The usefulness of Hb measured at hospital admission for detecting bleeding trauma patients is not clear in the literature and has been a topic of debate. In a retrospective cohort of 1000 trauma patients (140 of whom were in moderate to severe shock), Knottenbelt found that a low Hb level at hospital admission (mean of 2.6 h after injury) was related to the severity of hypotension and mortality [4]. In a retrospective study of 404 trauma patients (39 of whom required emergent hemostatic procedures), Bruns and colleagues [10] found that a Hb level ≤ 10 g/dl, measured using the HemoCue^®^ POC device within 30 min of the patient’s arrival at hospital, was associated with a threefold increase in the need for an emergent hemostatic procedure. Other authors found that Hb measured upon admission was not accurate in identifying major injuries [11, 12] or the need for an emergent hemostatic intervention [13, 14]. In the present study, Hb-Lab_hosp_ had a strong ability to predict SH (AUC of 0.92), but this measurement is taken in the laboratory and so results are subject to a delay. We showed that POC-Hb_hosp_, a quick Hb measurement taken immediately at hospital admission by using a POC device, performed equally well for predicting SH (AUC of 0.88) than Hb-Lab_hosp_. This is clinically relevant information since adequate resuscitative and hemostatic therapies should be implemented as soon as possible in the context of severe bleeding.

Beyond the static Hb measurements described above, the present study showed that DeltaPOC-Hb and the amount of prehospital fluid volume administered (FV_prehosp_) significantly predicted SH. Some authors have recently found that the amount of prehospital fluid volume administered was associated with hemodilution, coagulopathy and the need for massive transfusion [23, 24]. As far as we know, the present study provides for the first time a graphic description of the relationship between DeltaPOC-Hb and FV_prehosp_ in trauma patients with and without significant hemorrhage. We found that this relationship was significantly different between these two groups: for a given prehospital fluid volume infused, the magnitude of the drop in Hb was significantly higher in patients with significant hemorrhage than in controls. The relationship between the prehospital fluid infused and the drop of Hb is complex (Fig. 3 and Additional file 2). The “refilling” phenomenon (interstitial fluid movement into the vascular system) might occur early after the trauma (reflected in the lower POC-Hb value measured on scene in patients with SH compared to controls), and part of the infused volume might rapidly diffuse into the interstitial space, depending on both the inflammatory response and the endothelial lesions following injury. Further studies are necessary to better characterize the interactions between Hb loss, refilling, resuscitation-induced hemodilution and capillary leakage.

Several limitations of the present study must be acknowledged. First, only patients with at least one POC-Hb_prehosp_ measurement were included in the present study, representing a possible selection bias. Although this measurement was taken at the discretion of the prehospital medical team, this criterion allowed the inclusion of 74% of the total number of trauma patients admitted to the six centers during the study period. Moreover, 755 patients experienced SH within the 6402 trauma patients included, representing a very large cohort of bleeding trauma patients. The absence of a POC-Hb_prehosp_ was the only exclusion criterion. Excluded patients were compared to the study cohort, and only minor differences were noted: They were slightly older, the proportion of men was higher, and the frequency of SH was lower in comparison with the studied population (data not shown). The present study included all consecutive patients who were admitted to six level-1 trauma centers for suspicion of severe trauma, with a subsequent risk of overtriage. Indeed, some patients had low ISS in our cohort, and we did not exclude these patients from the statistical analysis to avoid any additional source of selection bias.

Another questionable point is the SH definition used in the present study. Various definitions of significant hemorrhage have been described in the literature including low arterial pressure [4], estimated blood loss [9], the need for hemostatic interventions [6, 10], transfusion requirements and/or massive transfusion [7, 8]. Massive transfusion is usually defined as 10 PRBCs in the first 24 h [25], but this definition is currently questioned [26, 27] and several other criteria have been shown to be better correlated with mortality: transfusion requirements in the first 6 h, transfusion of at least three PRBCs in 1 h or transfusion of five PRBCs in 4 h [27, 28]. The transfusion threshold used in the present study seems to represent a reasonable balance between those different definitions. Defining SH as the need for transfusion of 4 units of RBC in the first 6 h takes into account quantitative (half blood volume) and dynamic (time constraints) aspects of SH resuscitation, as suggested by some authors [29, 30]. Death by exsanguination in the first 24 h was chosen to allow the inclusion of the most severe patients. In the present study, comparable results were found by defining SH as “requirement of any hemostatic intervention following hospital admission” (data not shown). Since physicians were not blinded to the POC-Hb measurements, it could be argued that these values might have influenced the decision to transfuse. Nevertheless, transfusion was decided and performed only after hospital admission, whereas a POC-Hb measurement was taken by each prehospital and hospital team. Thus, this limitation does not seem to apply to the prehospital POC-Hb measurement. In addition, Hb-Lab_hosp_ had the best ability to predict SH although this result is available with delay for the trauma team, frequently once the decision to transfuse the first blood products has been taken. Following injury, the decision to initiate transfusion is not only based on a single Hb measurement, but rather by taking into account various factors such as clinical signs of shock, positive FAST (focused assessment with sonography for trauma) echography or obvious external hemorrhage, as it has recently been shown [31]. A last notable point is that the best POC-Hb_prehosp_ and POC-Hb_hosp_ cutoff values for predicting significant hemorrhage found in the present study are relatively high: respectively, 12 and 10 g/dl for women, 13 and 12 g/dl for men. These values are significantly higher than the routinely used threshold values of 7–9 g/dl recommended to trigger transfusion [3]. This finding indicates that active bleeding, and so the need for transfusion and/or hemostatic procedure, should be suspected before Hb decreases to these usual thresholds values.

## Conclusions

The first Hb measurement taken on scene by using a POC device was able to predict significant hemorrhage (SH) in our cohort of trauma patients. Even if this ability was modest (AUC = 0.72), our results challenge the common conception that the initial Hb value is not a reliable predictor of bleeding in trauma patients. POC-Hb_prehosp_ may be used alone (POC-Hb_prehosp_ < 12 g/dl had 90% specificity to predict SH) or with POC-Hb_hosp_ to determine DeltaPOC-Hb (POC-Hb_hosp_ − POC-Hb_prehosp_, best cutoff value of − 2 g/dl to predict SH). It could also be used in combination with some other variables, representing a valuable tool for the early identification of SH and subsequent implementation of adequate resuscitative and hemostatic therapies. POC-Hb_hosp_ had a greater ability to identify patients with SH than the widely used shock index, with a best cutoff value of 10 g/dl for women and 12 g/dl for men. The diagnostic performance of this variable was comparable with that of using Hb measured in the hospital laboratory. For a given prehospital fluid volume infused, the magnitude of the Hb drop was significantly higher in patients with a significant hemorrhage than in controls.

## Additional files

**Additional file 1.** Additional statistical analysis.

**Additional file 2.** Relationship between Hb drop (DeltaPOC-Hb) and prehospital fluid volume (FV_prehosp_) by using univariate stratified analysis.

## Abbreviations

AUC: area under the ROC curve
BMI: body mass index
BP: blood pressure
CI: confidence interval
CL: controls
CT scan: computed tomography scan
DeltaPOC-Hb: POC-Hb_hosp_ − POC-Hb_prehosp_
FFP: fresh frozen plasma
FV_prehosp_: total prehospital fluid volume administered to the patients
Hb: hemoglobin
Hb_hosp_: hemoglobin level provided by the laboratory at hospital admission
POC-Hb_prehosp_: prehospital hemoglobin level provided by point-of-care device
POC-Hb_hosp_: hospital hemoglobin level provided by point-of-care device
HR: heart rate
Hct: hematocrit
ICU: intensive care unit
ISS: injury severity score
Lactate_hosp_: serum lactate measured at hospital admission
MTP: massive transfusion protocol
NPV: negative predictive value
NS: nonsignificant
OR: operating room
PC: platelet concentrates
PLR: positive likelihood ratio
POC: point-of-care
PPV: positive predictive value
RBCs: red blood cells
ROC-curve: receiver operating characteristic curve
Se: sensitivity
SH: significant hemorrhage
SI: shock index (heart rate/systolic arterial pressure)
SI_hosp_: heart rate at hospital admission/systolic arterial blood pressure at hospital admission
SI_prehosp_: maximal prehospital heart rate/minimal prehospital systolic arterial blood pressure
Spe: specificity
U: units
Y: Youden
