# Supplementary material for: How useful are hemoglobin concentration and its variations to predict significant hemorrhage in the early phase of trauma? A multicentric cohort study
Source: Ann Intensive Care. 2018 Jul 6;8:76. doi: 10.1186/s13613-018-0420-8 (PMC6035120; doi:10.1186/s13613-018-0420-8)
Supplement: Supplementary file 1 — Additional file 1. Additional statistical analysis. [file 13613_2018_420_MOESM1_ESM.docx]

**Additional Table N°1.**

Multivariate logistic regression predicting significant hemorrhage (SH) during the prehospital period.

|  | **OR** | **95%CI** |
| --- | --- | --- |
| POC-Hb_prehosp_ | 0.73 | 0.70-0.73 |
| FV_prehosp_ | 1.01 | 1.00-1.02 |
| Shock index_prehosp_ | 2.22 | 1.90-2.61 |

All explicative variables were independently and significantly predictive of SH with p< 2e-16

**Additional Table N°2.** Multivariate logistic regression predicting significant hemorrhage (SH) on arrival at hospital.

|  | **OR** | **95%CI** |
| --- | --- | --- |
| POC-Hb_hosp_ | 0.02 | 0.01-0.03 |
| FV_prehosp_ | 1.01 | 1.00-1.02 |
| Shock index_hosp_ | 5.42 | 4.12-7.15 |
| Lactate_hosp_ | 1.25 | 1.20-1.30 |
| DeltaPOC-Hb | 0.95 | 0.89-1.00 |

All explicative variables were independently and significantly predictive of SH with p< 2e-16 except DeltaPOC-Hb (p=0.06).

**Additional Table N°3.**

Multivariate logistic regression predicting significant hemorrhage (SH) while adjusting on sex, age, hemoglobin drop (DeltaPOC-Hb) and on different levels of fluid volume (FV) therapy (qualitative variable with 5 levels: <500 ml, 500-1000ml, 1000-1500 mL, 1500 – 2000 mL and > 2000 mL).

|  | **OR** | **95%CI** |
| --- | --- | --- |
| FV_prehosp_ < 500 | 0.02 | 0.01-0.03 |
| FV_prehosp_ 500 – 1000 | 3.09 | 2.39-4.02 |
| FV_prehosp_ 1000 – 1500 | 6.14 | 4.65-8.14 |
| FV_prehosp_ 1500 – 2000 | 8.73 | 6.28-12.14 |
| FV_prehosp_ > 2000 | 18.10 | 12.49-26.35 |
| Gender | 0.71 | 0.57-0.88 |
| Age | 1.01 | 1.01-1.02 |
| DeltaPOC-Hb | 0.66 | 0.63-0.70 |

All explicative variables were independently and significantly predictive of SH with p< 2e-16 for fluid therapy and DeltaPOC-Hb, p<0.01 for the age and p<0.001 for gender.

The interaction term Fluid Volume infused * DeltaPOC-Hb was not significant in the model to predict severe hemorrhage.

**Additional Table N°4.**

Ranges and number of patients included in the grey zone.

| **Variable** | **Number of patients with significant hemorrhage**  **(Percentage of the study population)** | **Number of patients without significant hemorrhage**  **(Percentage of the study population)** |
| --- | --- | --- |
| POC-Hb_prehosp_ |  |  |
| < 12 g/dl | 344 (5) | 904 (14) |
| 12 - 15 g/dl | 367 (6) | 3434 (54) |
| > 15 g/dl | 44 (1) | 1309 (20) |
|  | Total = 755 (12) | Total = 5647 (88) |
| POC-Hb_hosp_ |  |  |
| < 11 g/dl | 564 (9) | 783 (12) |
| 11 - 13 g/dl | 98 (1.5) | 1049 (16) |
| > 13 g/dl | 93 (1.5) | 3815 (60) |
|  | Total = 755 (12) | Total = 5647 (88) |

**Additional Table N°5.**

Results obtained specifically in patients over 65-years-old.

|  | **Significant Hemorrhage**  **n = 106** | **Controls**  **n = 403** | **p value** |
| --- | --- | --- | --- |
| POC-Hb_prehosp_  (g dl^-1^) | 12 [11-13] | 13 [12-14] | < 0.001 |
| POC-Hb_hosp_  (g dl^-1^) | 9 [8-10] | 12 [11-14] | < 0.001 |
| DeltaPOC-Hb (g dl^-1^) | -3 [-4;-1] | -1 [-2;0] | < 0.001 |
| Hb-Lab_hosp_ (g dl^-1^) | 9 [7-10] | 12 [11-14] | < 0.001 |

**Additional Table N°6.**

Results obtained specifically in patients over 65-years-old.

| **Variable** | **AUC** | **Cut-off** | **Se (%)** | **Spe (%)** |
| --- | --- | --- | --- | --- |
| POC-Hb_prehosp_ (g dl^-1^) | 0.65 | 12 | 62 | 62 |
| POC-Hb_hosp_ (g dl^-1^) | 0.85 | 10 | 74 | 80 |
| DeltaPOC-Hb (g dl^-1^) | 0.77 | -2 | 59 | 83 |
| Hb-Lab_hosp_ (g dl^-1^) | 0.90 | 10 | 85 | 79 |
