# Supplementary material for: How useful are hemoglobin concentration and its variations to predict significant hemorrhage in the early phase of trauma? A multicentric cohort study
Source: Ann Intensive Care. 2018 Jul 6;8:76. doi: 10.1186/s13613-018-0420-8 (PMC6035120; doi:10.1186/s13613-018-0420-8)
Supplement: Supplementary file 2 — Additional file 2. Relationship between Hb drop (DeltaPOC-Hb) and prehospital fluid volume (FVprehosp) by using univariate stratified analysis. [file 13613_2018_420_MOESM2_ESM.pptx]

## Slide 1
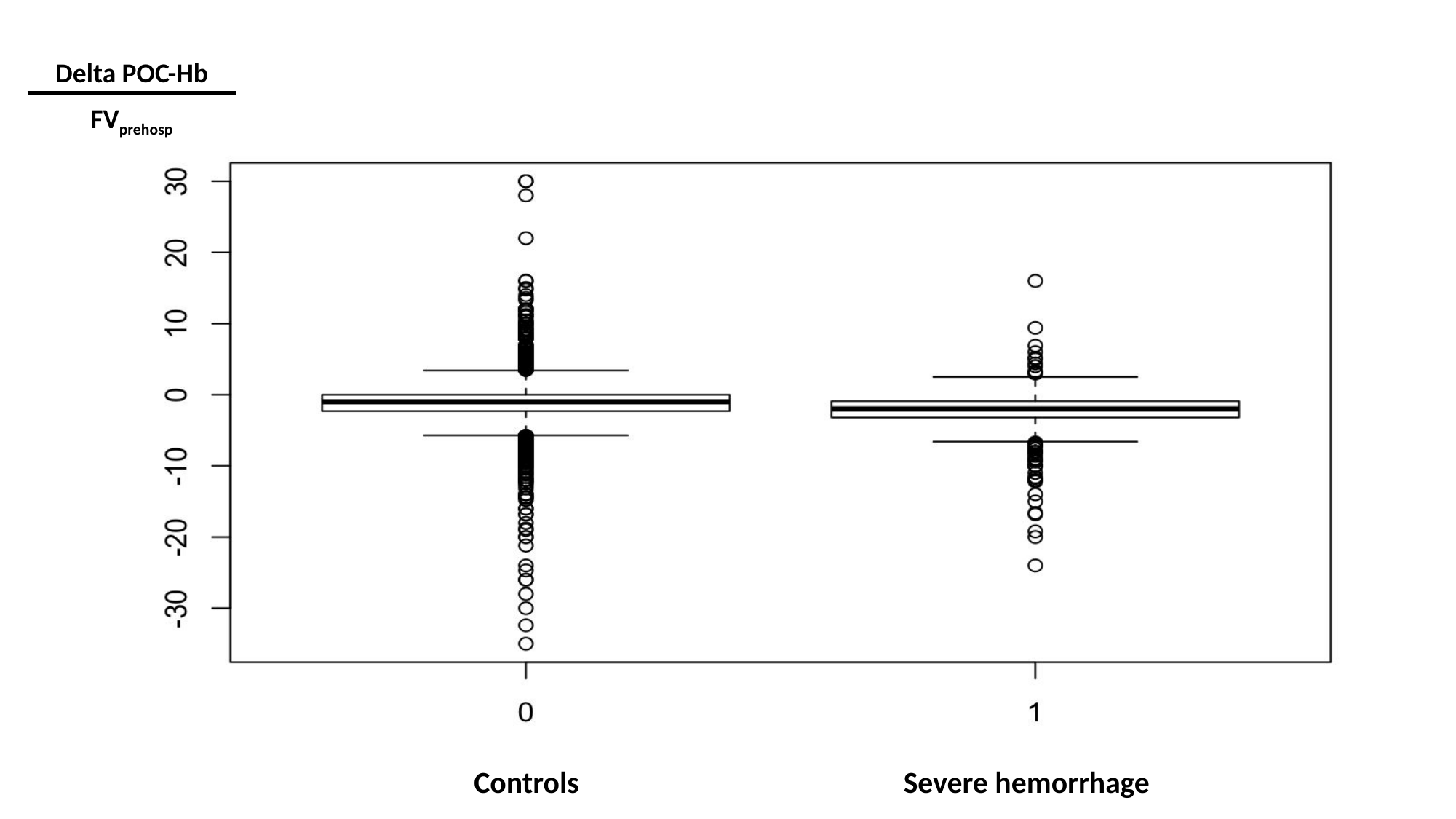

Delta POC-Hb
FVprehosp
Controls
Severe hemorrhage

## Slide 2
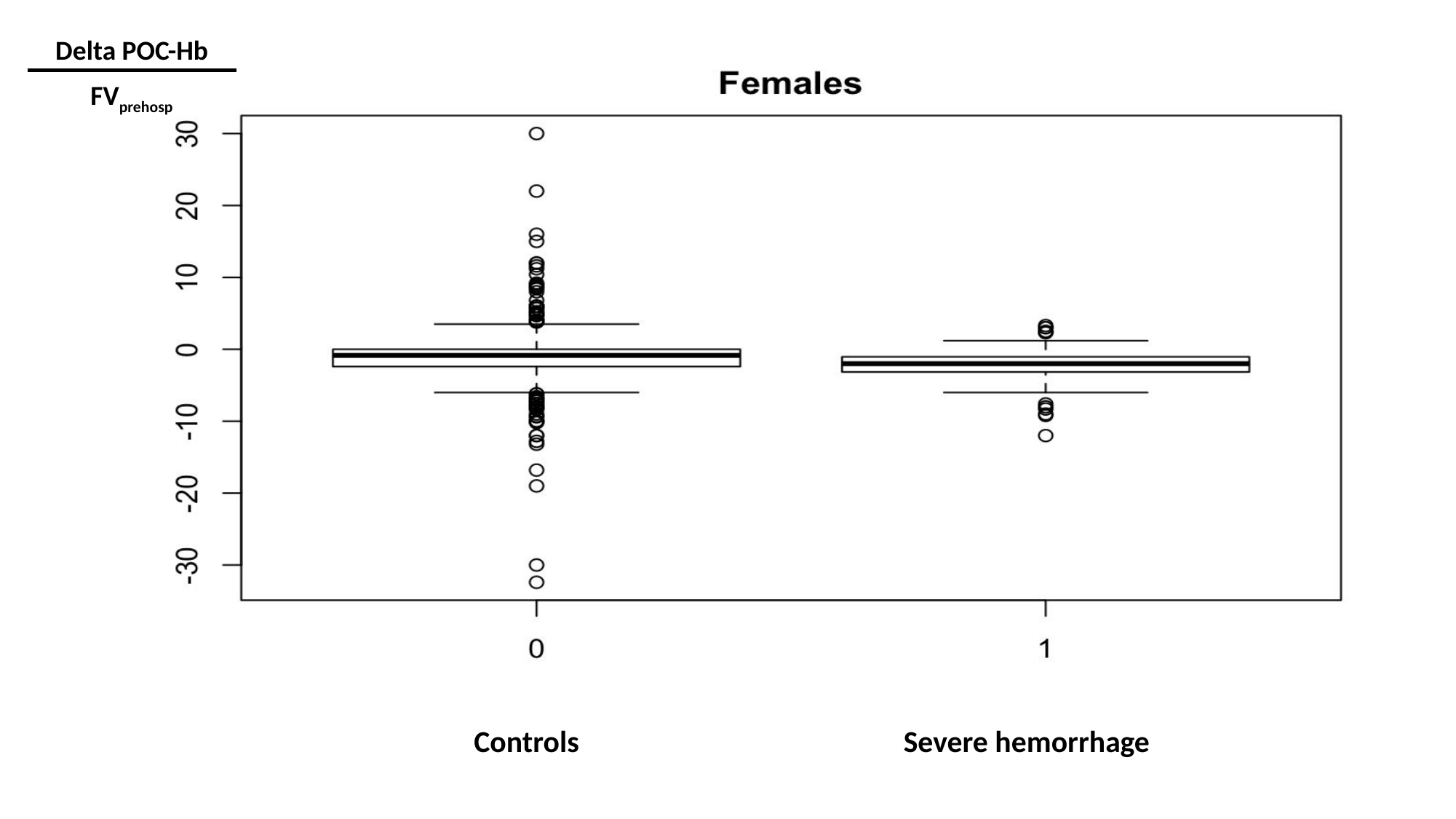

Delta POC-Hb
FVprehosp
Controls
Severe hemorrhage

## Slide 3
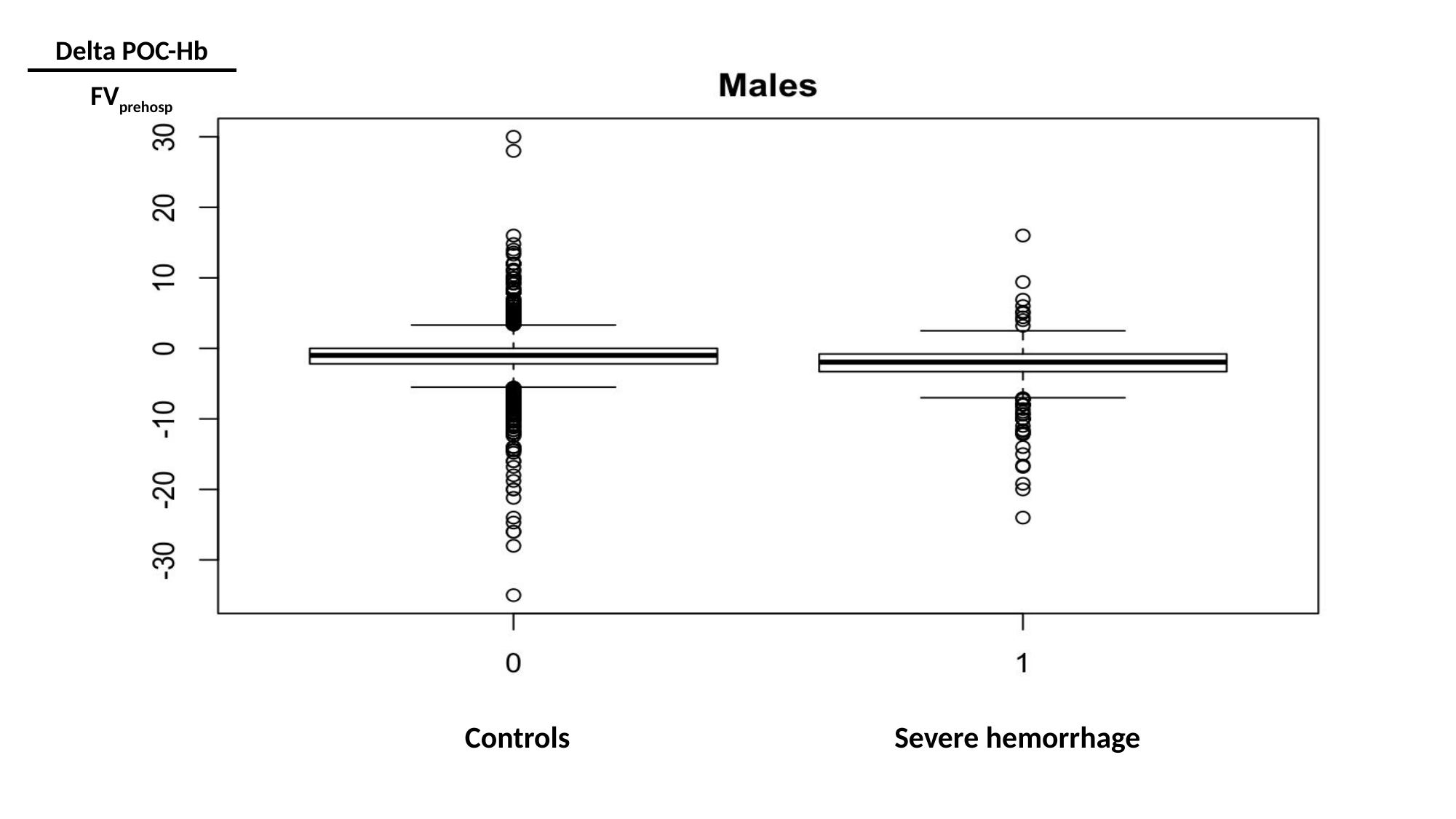

Delta POC-Hb
FVprehosp
Controls
Severe hemorrhage
